# Re: The role of ^68^Ga-PSMA-PET/CT in radiotherapy planning in prostate cancer

**DOI:** 10.1590/S1677-5538.IBJU.2019.0130

**Published:** 2019-09-02

**Authors:** Yasemin Benderli Cihan

**Affiliations:** 1 Kayseri Education and Research Hospital, Department of Radiation Oncology, Turkey

To the editor,

Prostate cancer is the second most common cancer in men worldwide. The incidence increases above 50 years. Although prostate cancer is observed in more than 80% of males over 80 years of age, most patients have a slow course and no clinical symptoms. As with all malignancies, early diagnosis is important in prostate cancer. The most important diagnostic methods used in prostate cancer are digital rectal examination, transrectal ultrasonography and PSA. The definitive diagnosis is made by histopathology. Nowadays, it is diagnosed at localized stage (1-4).

The correct staging of the disease after the diagnosis of prostate cancer is very important in terms of the effectiveness and prognosis of treatment planning. In the staging, thoraco-abdominal Computed Tomography (CT), pelvic Magnetic Resonance Imaging (MRI), bone scintigraphy (TVKS), NaF-PET / CT imaging and ^68^Ga-PSMA-PET/CT imaging, which are currently used as a new diagnostic method, are used. Fluorine-18 (2-fluoro-2-deoxy D-glucose) used in 18F-FDG PET / CT is generally not used in staging because of its low or no involvement in prostate cancer (1-3).

Although the role of ^68^Ga-PSMA-PET/CT in the management of prostate cancer has not yet taken place in the international guidelines, in the recent studies ^68^Ga-PSMA-PET/CT staging, treatment plan determination, suspicion of recurrence or biochemical recurrence in the presence of existing lesions has a significant contribution to the evaluation of the response to treatment with high diagnostic accuracy. PSMA is a type II transmembrane protein which increases expression in prostate cancer cells. This can be accurately detected in prostate cancer cells by ^68^Ga-PSMA-PET/CT examination. However, since 5% of patients with prostate cancer may not have PSMA expression, a false negative result can be obtained with ^68^Ga-PSMA-PET/CT examination (3-6). In the study performed by Eiber et al., the PSA value of ^68^Ga-PSMA-PET/CT was 2ng / dl or above, 96,8%, 93% between 1-2, 72% between 0,5-1 and 0,2-0,5. 57.9% of the patients had diagnostic accuracy. ^68^Ga-PSMA-PET/CT was reported to have high diagnostic accuracy in lesion detection even at low PSA (less than 0.5) (2). Maurer et al. compared the mid-high-risk group with ^68^Ga-PSMA-PET/CT and conventional radiological imaging (CT or MRI) in nodal smear diagnosed with prostate cancer (130 patients). According to the data obtained, the sensitivity, specificity and diagnostic accuracy of ^68^Ga-PSMA-PET/CT were 65.9%, 98.9% and 88.5%, respectively, whereas these rates were 43.9%, 85.4% and 72.3%, respectively. The diagnostic accuracy of ^68^Ga-PSMA-PET/CT was reported to be higher in nodal staging (3). Pyka et al. reported that ^68^Ga-PSMA-PET/CT was more sensitive and specific in demonstrating bone metastasis in a study of ^68^ patients with prostate cancer compared with TVKS findings and 6 ^68^Ga-PSMA-PET/CT findings (4).

Both primary and definite radiotherapy (RT) can change the target area in salvage RT. Schmidt-Hegemann et al. examined the reliability of the ^68^Ga-PSMA-PET / CT, the treatment plan in many patients; both primary definitive RT and salvage can change the target area in RT. Compared with conventional CT, ^68^Ga-PSMA-PET/CT reported a significant effect on the radiotherapeutic approach, especially in postoperative patients (5). In a prospective study, Roach et al. evaluated ^68^Ga-PSMA-PET/CT images for restaging because of suspicion of recurrence or biochemical recurrence in the middle and high risk group. They found that 51% of the patients’ treatment plan changed when they evaluated the treatment plan with before and after the treatment plan. This rate was found to be 21% for primary staging. As a result, ^68^Ga-PSMA-PET/CT reported higher sensitivity in detecting biochemical recurrence (6). In another study, 68Ga-PSMA-PET / CT and CT were compared in patients with salvage radiotherapy. It has been reported that 28.6% more pathological involvement is detected in ^68^Ga-PSMA-PET/CT , CT, and ^68^Ga-PSMA-PET/CT has been reported to be more useful in determining RT volume and indication (7). Zamboglou et al. compared ^68^Ga-PSMA-PET/CT with MRI for the detection of gross tumor volume (GTV) in primary prostate cancer. It was reported that ^68^Ga-PSMA-PET/CT may play an important role in focal radiation planning in lesions (8).

As a result, ^68^Ga-PSMA-PET/CT is a more frequently used test because of its high specificity and sensitivity in detecting recurrent lesion and localization even in low PSA levels. In addition, the metabolic activity of the primary site and the local area in the patient with staging as well as the RT plan provides valuable advantages about the width of the RT area. According to all the findings, prospective studies are needed to determine the role and importance of ^68^Ga-PSMA-PET/CT in RT plan in prostate cancer.

Yours Sincerely,

Author
